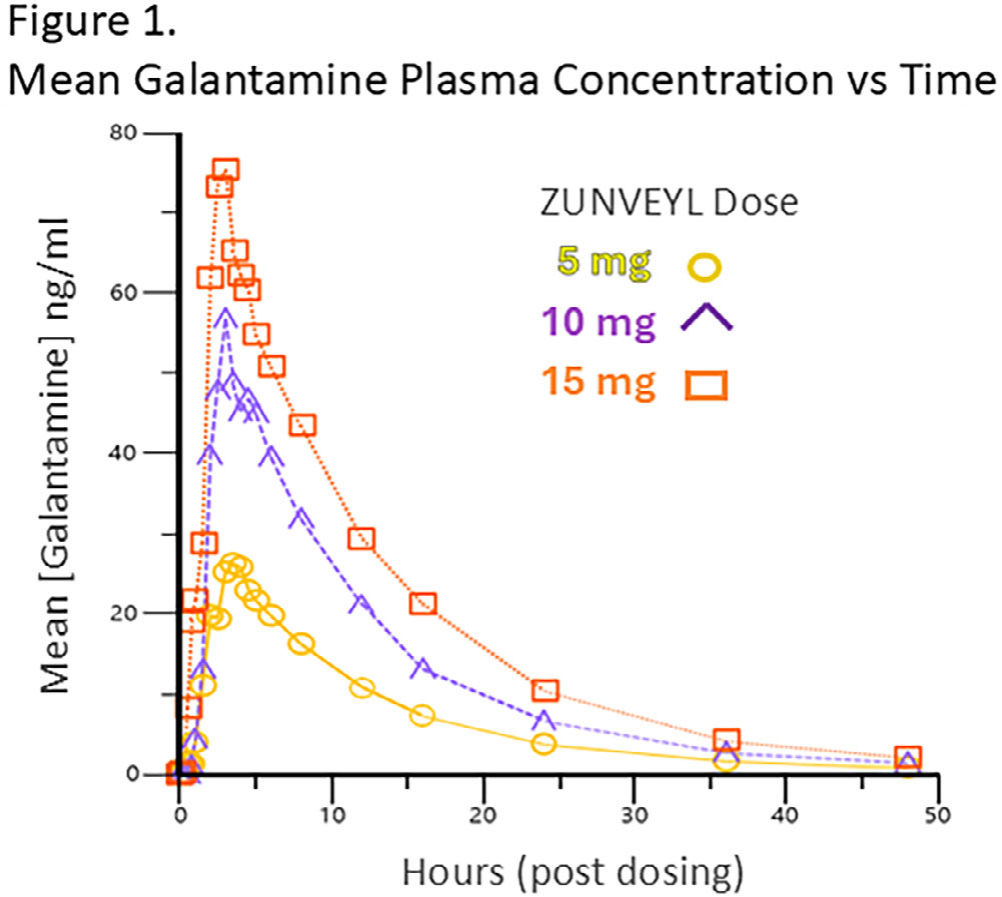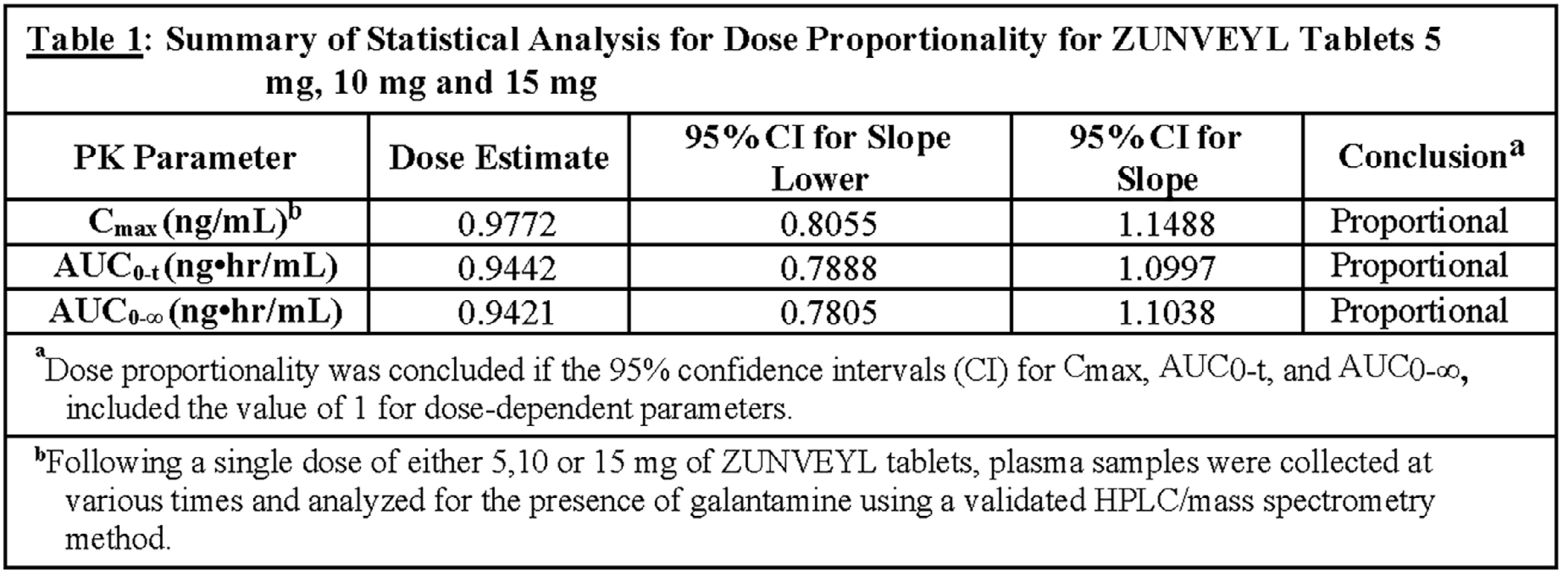# Dose Proportionality of ZUNVEYL a Galantamine Prodrug Over a Dosage Range of Five to Fifteen Milligrams Demonstrated Under Fasting Conditions

**DOI:** 10.1002/alz70859_107255

**Published:** 2025-12-26

**Authors:** Denis G Kay, Kurt P Grady, Andrew J Wahlert, Michael McFadden

**Affiliations:** ^1^ Alpha Cognition Inc, Vancouver, BC Canada

## Abstract

**Background:**

ZUNVEYL (benzgalantamine, galantamine benzoate gluconate), is a pharmacologically inactive prodrug of galantamine. ZUNVEYL was FDA approved in 2024 for BID dosing for the treatment of mild to moderate Alzheimer’s dementia, via the 505(b)(2) regulatory pathway, relying on FDA’s previous finding of safety and efficacy for the listed drugs (LDs) Razadyne® (galantamine hydrobromide tablets), and Razadyne® ER (galantamine hydrobromide extended‐release capsules). When dosed as a delayed‐release (DR) tablet, ZUNVEYL bypasses the stomach and, is absorbed in the small intestine potentially reducing the gastrointestinal side effects common for acetylcholinesterase inhibitors. Consequently, ZUNVEYL may offer advantages over the other acetylcholinesterase inhibitors by reducing gastrointestinal adverse effects which limit patient compliance. Here we report the outcome of a study designed to assess the dose proportionality of ZUNVEYL DR tablets over a dosage range of 5‐15 milligrams.

**Methods:**

This was an open‐label, balanced, randomized, three‐arm, three‐treatment, single‐dose parallel study to evaluate the pharmacokinetics and dose proportionality of ZUNVEYL DR tablets of 5, 10 and 15 mg strength in healthy adult subjects (N=42), under fasting conditions. The study protocol underwent ethics review and was conducted under GCP conditions.

**Results:**

The dose proportionality of ZUNVEYL based on the plasma analysis of galantamine derived from ZUNVEYL (Table 1), and the pharmacokinetic profiles of all three doses (Figure 1) are presented below. Fifteen milligrams of ZUNVEYL administered to drug naïve subjects, provoked one gastrointestinal adverse event (1/14), a lower incidence than that expected with an equivalent dose of Galantamine hydrobromide.

**Conclusions:**

ZUNVEYL was well‐tolerated, no serious adverse events were observed. Statistical analysis confirmed the dose proportionality for Cmax, AUC0‐t, & AUC0‐∞ over the entire 5 mg to 15 mg dose range for ZUNVEYL. The studies demonstrating bioequivalence, under fed and fasted (abstract #107030), and steady state (abstract #107147) conditions, and dose proportionality studies, lead to the conclusion that one ZUNVEYL 5 mg DR tablet is equivalent to one galantamine hydrobromide 4 mg immediate release tablet.